# Species Differences in the Immunoreactive Expression of Oxytocin, Vasopressin, Tyrosine Hydroxylase and Estrogen Receptor Alpha in the Brain of Mongolian Gerbils (*Meriones unguiculatus*) and Chinese Striped Hamsters (*Cricetulus barabensis*)

**DOI:** 10.1371/journal.pone.0065807

**Published:** 2013-06-07

**Authors:** Yu Wang, Linxi Xu, Yongliang Pan, Zuoxin Wang, Zhibin Zhang

**Affiliations:** 1 State Key Laboratory of Integrated Management of Pest Insects and Rodents in Agriculture, Institute of Zoology, Chinese Academy of Sciences, Beijing, PR China; 2 University of Chinese Academy of Sciences, Beijing, PR China; 3 Department of Psychology and Program in Neuroscience, Florida State University, Tallahassee, Florida, United States of America; University of Texas Health Science Center at San Antonio, United States of America

## Abstract

Species differences in neurochemical expression and activity in the brain may play an important role in species-specific patterns of social behavior. In the present study, we used immunoreactive (ir) labeling to compare the regional density of cells containing oxytocin (OT), vasopressin (AVP), tyrosine hydroxylase (TH), or estrogen receptor alpha (ERα) staining in the brains of social Mongolian gerbils (*Meriones unguiculatus*) and solitary Chinese striped hamsters (*Cricetulus barabensis*). Multiple region- and neurochemical-specific species differences were found. In the anterior hypothalamus (AH), Mongolian gerbils had higher densities of AVP-ir and ERα-ir cells than Chinese striped hamsters. In the lateral hypothalamus (LH), Mongolian gerbils also had higher densities of AVP-ir and TH-ir cells, but a lower density of OT-ir cells, than Chinese striped hamsters. Furthermore, in the anterior nucleus of the medial preoptic area (MPOAa), Mongolian gerbils had higher densities of OT-ir and AVP-ir cells than Chinese striped hamsters, and an opposite pattern was found in the posterior nucleus of the MPOA (MPOAp). Some sex differences were also observed. Females of both species had higher densities of TH-ir cells in the MPOAa and of OT-ir cells in the intermediate nucleus of the MPOA (MPOAi) than males. Given the role of these neurochemicals in social behaviors, our data provide additional evidence to support the notion that species-specific patterns of neurochemical expression in the brain may be involved in species differences in social behaviors associated with different life strategies.

## Introduction

Animals show remarkable differences in their life strategies and social behaviors. Social species, for example, usually display high levels of prosocial behavior towards conspecifics, social affiliation with mates and biparental care of their offspring [1], [2]. In contrast, solitary species generally display low levels of prosocial behavior and social affiliation, but high levels of aggression to defend their territory [1], [3]. Such species differences in life strategy and social behavior may not only reflect their adaption to the environment, but also indicate their potential differences in the central mechanisms that are involved in the regulation of social behavior.

Indeed, several neurochemicals have been implicated in social behaviors associated with different life strategies. For example, the receptor distribution and activity of the neuropeptides oxytocin (OT) and vasopressin (AVP) differ in the brains of social and nonsocial rodent species, and such differences are thought to be involved in the regulation of species-specific social behaviors, such as affiliation, pair bonding, male parental care and territory marking [4]–[7]. Similarly, release patterns of the neurotransmitter dopamine, during mating and social interaction, differ between social and nonsocial rodent species [7], and dopamine has been implicated in pair bonding behavior in the socially monogamous rodent species [8]–[10]. The distribution patterns of estrogen receptor alpha (ERα) in the brain also differ between social and nonsocial rodent species [11], [12], and ERα has been implicated in social behaviors such as social affiliation, aggression and maternal care [13]–[17]. Interestingly, ERα may regulate social behaviors by interacting with other neurotransmitter systems [18], [19]. For instance, ERα may affect AVP expression in certain brain areas [20], [21] and alters AVP-mediated behaviors, such as aggression and affiliation [22], [23].

Many comparative studies have focused on species that share a close phylogenetic relationship and that are even in the same genus [1], [6], [24]–[28]. We have expanded our efforts to examine species from different genera, as these data will help us to better understand how evolution shapes the brain and behavior. In a most recent study, we compared social Brandt's voles (*Lasiopodomys brandtii*) with solitary Greater long-tailed hamsters (*Tscherskia triton*), and found species differences in central OT and AVP immunoreactive (ir) staining in brain areas important for social behaviors [2]. These data support the notion that central OT and AVP may underlie species differences in social behaviors [1], [2], [25], [26], [29]. In the present study, we extended our efforts to additional rodent species, particularly Mongolian gerbils (*Meriones unguiculatus*) and Chinese striped hamsters (*Cricetulus barabensis*), to further test the hypothesis that differences in neurochemical systems in the brain are related to species differences in life strategies and behaviors. In addition to central OT and AVP systems, we included TH (a dopamine-related marker) and ERα in our investigation, as both have been implicated in social behaviors [7], [14]–[16].

Mongolian gerbils inhabit typical steppes in Inner Mongolia and in the south-east of the Bakal area in Russia and Mongolia [30]–[32]. These animals are diurnal and highly social. They live in large family groups that range from 2 to 17 individuals and usually consist of a breeding male and female, as well as the siblings and offspring of the breeding pair [33], [34]. Extensive social interactions have been observed among individuals [33], [35]. Females and males form monogamous pairs in nature [33], [34], [36] and both parents play an active role in nest building and caring for offspring [37]. Furthermore, female Mongolian gerbils display higher levels of parental care and food hoarding behavior compared to male conspecifics whereas males show higher levels of territorial and aggressive behavior than females [34], [36], [38]–[40]. These gerbils become sexually mature around 5 months of age, and their life span is about 1 year in the wild and 2.5 years in the laboratory [33], [35]. In contrast, Chinese striped hamsters are primarily found in the farmland or grassland of northern China [41]. They are nocturnal and solitary, and display high levels of aggressive behavior towards conspecifics [41], [42]. In Chinese striped hamsters, females raise pups alone, and they also display higher levels of aggressive behavior compared to males [42]. Chinese striped hamsters reach sexual maturity around 3 months of age, and their life span is about 10 months [41].

In the present study, we compared OT, AVP, TH, and ERα immunoreactivity between these two species in selected brain areas known to be important in social behaviors. Such species differences in neurochemical expression in the brain may be ultimately involved in the regulation of species-specific social behaviors.

## Methods

### Ethics statement

The project was officially approved by the Institute of Zoology (IOZ), Chinese Academy of Sciences. People involved in caring and handling experimental animals were trained, and procedures related to animal care and ethics were approved by the examination of Animal Ethics Committee of IOZ (Permit Number: IOZ13002).

### Subjects

Subjects were adult male and female Mongolian gerbils (*Meriones unguiculatus*) and Chinese striped hamsters (*Cricetulus barabensis*). Mongolian gerbils were the offspring of a laboratory breeding colony that was maintained in the IOZ at the Chinese Academy of Sciences in Beijing, China. Chinese striped hamsters were captured in croplands nearby Qufu, Shandong Province in the winter of 2009. These animals were captured in private farms. Because Chinese striped hamster is a pest species, our capture got owner's permission and support. This capture does not harm other endangered species or rare species which are present in the farmland. We conducted trapping using steel-wire live traps (12 L×12 W×25 H cm). Fresh peanuts were used as bait, small pieces of cabbage were provided as a water supply and local dry leaves were provided as nest material. An iron sheet was attached on the upper side of the trap as shelter to protect from predation and sunshine. Pregnant and lactating females were released immediately on site. Captured animals were carefully transferred to the laboratory using the live trap. These animals were housed in the lab environment for about two weeks before they were used in the experiment. All subjects were housed in plastic cages (27 L×16 W×13 H cm for Mongolian gerbils and 25 L×14 W×14 H cm for Chinese striped hamsters) that contained wood shavings. Food and water were provided *ad libitum*. As Mongolian gerbils are social animals, they were housed in same-sex groups, consisting of two to four individuals, under a 16L:8D photoperiod (lights on 0500). Chinese striped hamsters are solitary animals and thus they were housed singly under a reversed 16L:8D photoperiod (lights on 1700). Room temperature was maintained at 20±2°C.

### Tissue preparation

Eight male and eight female Mongolian gerbils, and seven male and seven female Chinese striped hamsters were deeply anesthetized with sodium pentobarbital (3 mg/100 g body weight, Sigma-Aldrich, St. Louis, MO, USA) and perfused through the ascending aorta with 0.1 M phosphate buffered solution (PBS, pH7.2) followed by 4% paraformaldehyde in PBS. Brains were quickly removed, post-fixed in 4% paraformaldehyde for 12 h and then stored in 30% sucrose in PBS. Coronal brain sections (40 µm thickness) were cut on a cryostat. Four alternate sets of brain sections at 240 µm intervals were processed for OT, AVP, TH and ERα immunocytochemistry, respectively. An additional set of brain sections was processed for Nissl staining.

### Immunocytochemistry for OT, AVP, TH and ERα

Floating brain sections were processed for OT, AVP, or TH immunocytochemistry using an established method [28]. Briefly, brain sections were pre-treated with 0.5% NaBH_4_, followed by 0.05% H_2_O_2_ in 0.05 M Tris–NaCl (pH7.6), and then blocked in 10% normal goat serum (NGS) in Tris–NaCl with 0.5% Triton X-100 (Tris–Triton). Sections were incubated in rabbit anti-OT serum (1∶20,000), guinea pig anti-AVP serum (1∶40,000),or rabbit anti-TH serum (1∶15,000) (from Bachem California, Inc., Torrance, CA, USA), respectively, in Tris–Triton with 2% NGS for 36 h at 4°C, followed by an additional 2 h at room temperature. Thereafter, sections were incubated with a biotinylated goat-anti-rabbit, goat-anti-guinea pig or goat-anti-rabbit secondary antibody for OT, AVP and TH (1∶300; all from Vector Laboratories Inc., Burlingtone, CA, USA), respectively, in Tris–Triton for 2 h; ABC complex (Vector Laboratories Inc., Burlingtone, CA, USA) in Tris–NaCl for 90 min; and stained by 0.05% 3-3′-diaminobenzidine (Sigma-Aldrich) in Tris–NaCl with 0.009% H_2_O_2_. Sections were then mounted, air-dried and cover slipped.

The ERα immunocytochemistry was also conducted using an established method [43], [44]. Briefly, brain sections were pre-treated with 10 mM citrate buffer for 10 min at 90°C, followed by 0.5% NaBH_4_ for 5 min and then 0.5% H_2_O_2_ in 0.1 M PBS for 30 min. Thereafter, sections were treated in PBS with 0.6% Triton X-100 (PBT) for 20 min, blocked in 10% NGS in PBT for 30 min and incubated in rabbit ERα polyclonal antibody (1∶8000, Upstate, Millipore, Billerica, MA, USA) in PBT with 2% NGS for 36 h at 4°C and an additional 1 h at room temperature. Sections were then incubated with biotinylated goat-anti-rabbit secondary antibody (1∶300, Vector Laboratories Inc., Burlingtone, CA, USA) in PBT for 2 h, ABC complex in PBS for 90 min and stained by nickel-DAB. Sections were mounted, air-dried and cover slipped.

Brain sections for each neurochemical marker were processed concurrently to reduce variability in the staining. To control for antibody specificities, additional brain sections were incubated either in the absence of the primary antibody or with the primary antibody that was pretreated with 50 µM of OT, AVP, dopamine and estrogen, respectively. In these situations, specific staining was eliminated or substantially reduced.

### Data quantification and analysis

All slides were coded to conceal group identity. Slides were inspected under a Nikon microscope to identify forebrain regions quantified. OT-ir and AVP-ir cells were counted in the anterior hypothalamus (AH), lateral hypothalamic area (LH), medial preoptic area (MPOA) and paraventricular nucleus of the hypothalamus (PVN). TH-ir cells were counted in the AH, LH, MPOA, PVN, ventral tegmental area (VTA) and substantianigra pars compacta (SNc). ERα-ir cells were counted in the AH, MPOA, PVN, lateral septum (LS), bed nucleus of the striateminalis (BST), ventromedial hypothalamus (VMH), arcuate nucleus of the hypothalamus (ARC) and medial (MeA) and anterior cortical (CoA) nuclei of the amygdala. These brain areas were chosen based on previous studies from other rodent species indicating the existence of these neurochemicals and their potential roles in social behaviors.

Brain sections were matched between animals and 2–3 sections per brain area per animal were examined. Cells stained for each neurochemical marker within each brain area were quantified bilaterally. Further, a set of Nissl stained brain sections from each species was used to identify and measure the brain areas, which were then used to convert cell counts into cell density per area. Data were analyzed by a two-way analysis of variance (ANOVA) with species and sex as between-subject variables. Significant interactions were further evaluated by a Student-Newman-Keuls (SNK) post-hoc test. The criterion for significance was set at *p*<0.05.

## Results

### OT-ir staining

**Figure 1:**
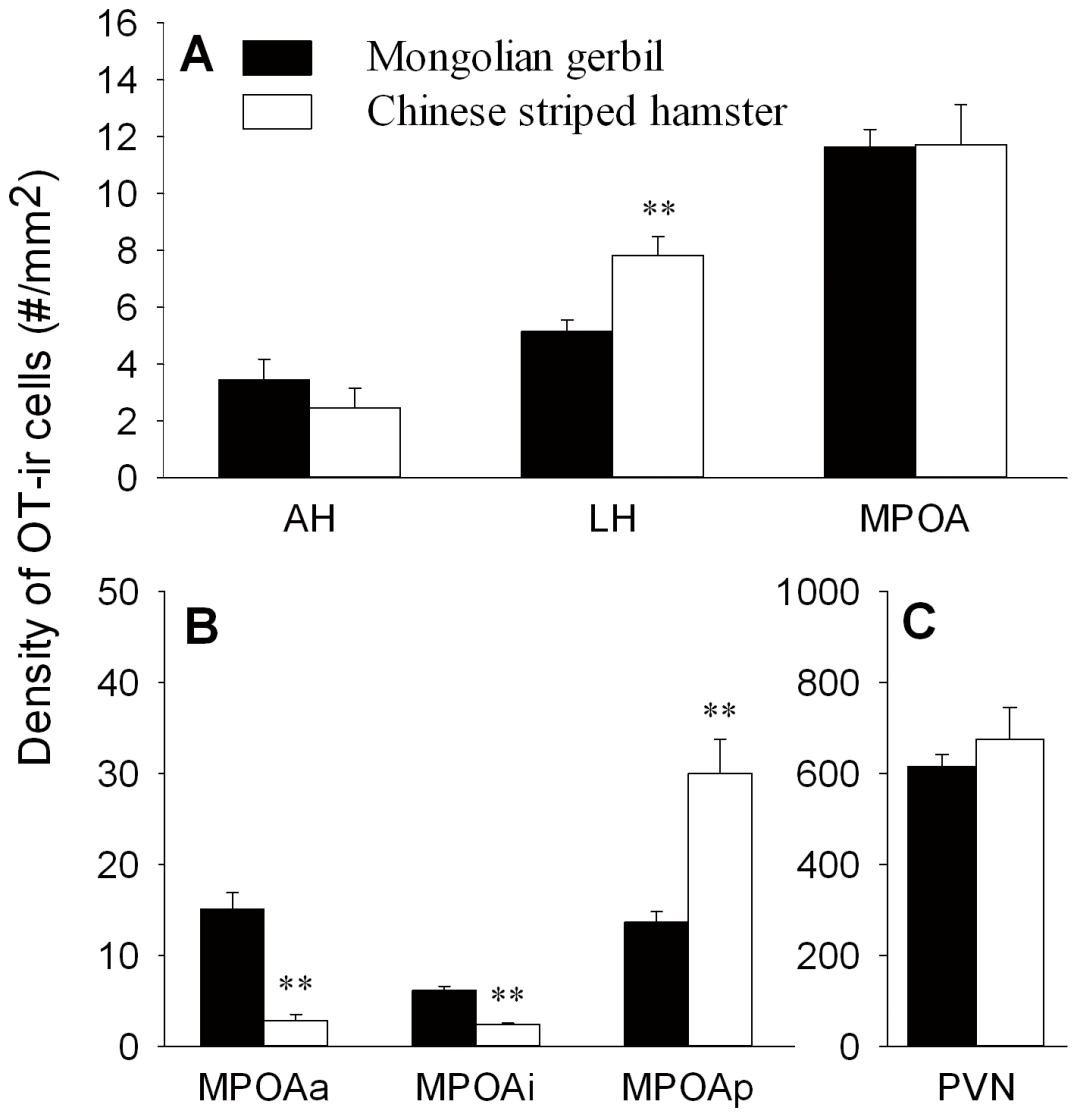
Species differences in the density of OT-ir cells in brain areas. (A) Species differences in the density of OT-ir cells in the anterior hypothalamus (AH), lateral hypothalamus (LH) and medial preoptic area (MPOA). (B) The two species also differed in the density of OT-ir cells in the subnuclei of the MPOA, including the anterior (MPOAa), intermediate (MPOAi) and posterior (MPOAp) part of the MPOA. (C) Species difference in the density of OT-ir cells in the paraventricular nucleus of the hypothalamus (PVN). ** *p*<0.01.

In both species, OT-ir stained cells were present either in dense clusters or scattered throughout many forebrain areas. Very intense staining of OT-ir cells was found in the PVN, while moderate clusters of OT-ir cells were present throughout the rostral–caudal extent of the MPOA. Scattered OT-ir cells were found in many brain regions including the AH and LH. Quantification of OT-ir cells in the above-mentioned brain areas (Table 1) indicates that Mongolian gerbils had a lower density of OT-ir cells in the LH than Chinese striped hamsters (F_(1,26)_ = 11.98, *p*<0.01), whereas no species differences were found in the AH, MPOA or PVN (Fig. 1). Although the two species showed similar densities of OT-ir cells in the MPOA, some differences were found in the subnuclei of the MPOA. Mongolian gerbils had a higher density of OT-ir cells in the anterior (MPOAa; F_(1,26)_ = 33.49, *p*<0.01) and intermediate (MPOAi; F_(1,26)_ = 75.64, *p*<0.01) nuclei of the MPOA than Chinese striped hamsters, whereas an opposite pattern was found in the posterior nucleus of the MPOA (MPOAp; F_(1,26)_ = 17.74, *p*<0.01) (Fig. 1B, Table 1). A sex difference was found in the OT-ir cell density in the MPOAi, in which females had a higher density of OT-ir cells than males (F_(1,26)_ = 7.50, *p*<0.05) (Table 1). No sex or species-sex interaction was found in any other brain areas examined.

**Table 1:** Density of oxytocin immunoreactive cells (mean±SEM/mm^2^) in the brain of male and female Mongolian gerbils and Chinese striped hamsters.

Brain area	Mongolian gerbil		Chinese hamster		Two-way ANOVA		
	Male	Female	Male	Female	species	sex	sp X sex
AH	2.4±0.7	4.5±1.3	2.1±0.6	2.8±1.3	ns	ns	ns
LH	5.7±0.6	4.6±1.0	7.8±1.0	7.8±1.0	**	ns	ns
MPOA	11.4±1.0	11.9±0.8	11.2±1.6	12.2±2.4	ns	ns	ns
MPOAa	15.4±2.6	14.8±2.7	2.1±0.9	3.6±1.1	**	ns	ns
MPOAi	5.1±0.5	7.2±0.6	2.2±0.2	2.6±0.2	**	*	ns
MPOAp	13.6±1.6	13.8±1.6	29.3±4.7	30.7±6.4	**	ns	ns
PVN	646.2±32.9	583.0±62.1	696.7±129.6	654.7±62.1	ns	ns	ns

SEM: standard error of the mean;

* *p*<0.05;

** *p*<0.01;

ns, not significantly different.

### AVP-ir staining

**Figure 2:**
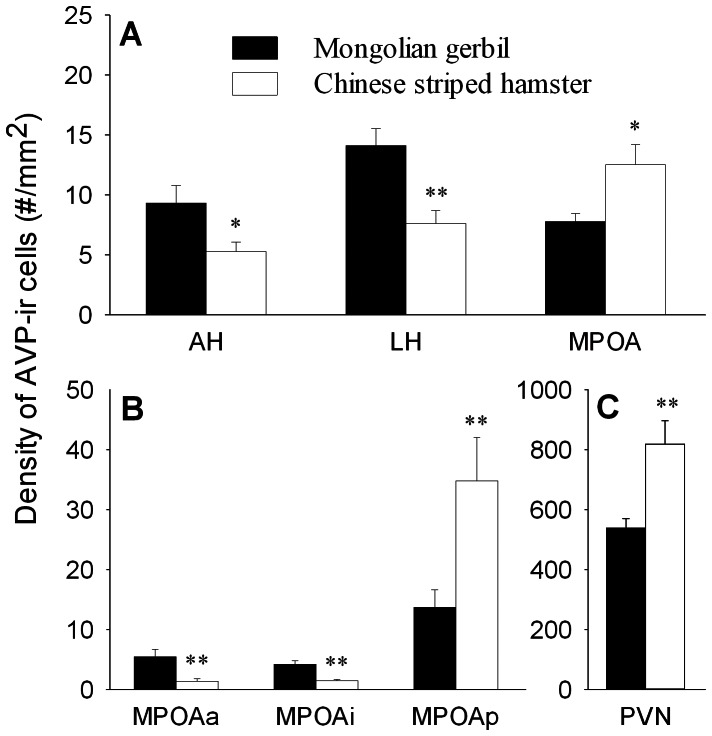
Species differences in the density of AVP-ir cells in brain areas. (A) Species differences in the density of AVP-ir cells in the anterior hypothalamus (AH), lateral hypothalamus (LH) and medial preoptic area (MPOA). (B) The two species also differed in the density of AVP-ir cells in the subnuclei of the MPOA, including the anterior (MPOAa), intermediate (MPOAi) and posterior (MPOAp) part of the MPOA. (C) A species difference was also found in the paraventricular nucleus of the hypothalamus (PVN). * *p*<0.05, ** *p*<0.01.

A dense cluster of AVP-ir cells was found in the PVN, moderate clusters of AVP-ir cells were found in the MPOA, mostly in the MPOAp, and scattered AVP-ir cells were observed in the AH and LH. Species differences were found in the density of AVP-ir cells in selected brain areas. Mongolian gerbils had a higher density of AVP-ir cells in the AH (F_(1,26)_ = 6.43,*p*<0.05) and LH (F_(1,26)_ = 12.50,*p*<0.01), but a lower density of AVP-ir cells in the MPOA (F_(1,26)_ = 7.29,*p*<0.05) and PVN (F_(1,26)_  = 11.59, *p*<0.01), than Chinese striped hamsters (Table 2, Fig. 2A & C). Within the MPOA, Mongolian gerbils had a higher density of AVP-ir cells in the MPOAa (F_(1,26)_ = 16.87, *p*<0.01) and MPOAi (F_(1,26)_ = 31.45, *p*<0.01) than Chinese striped hamsters, while an opposite pattern was found in the MPOAp (F_(1,26)_ = 16.34, *p*<0.01) (Fig. 2B, Table 2). No sex or species-sex interaction was found in the density of AVP-ir cells in any brain areas examined.

**Table 2:** Density of vasopressin immunoreactive cells (mean±SEM/mm^2^) in the brain of male and female Mongolian gerbils and Chinese striped hamsters.

Brain area	Mongolian gerbil		Chinese hamster		Two-way ANOVA		
	Male	Female	Male	Female	species	sex	sp X sex
AH	6.4±1.2	12.3±2.3	5.5±1.3	5.0±1.0	*	ns	ns
LH	15.4±1.6	12.9±2.4	7.0±1.8	8.2±1.3	**	ns	ns
MPOA	7.7±1.0	7.9±1.0	11.4±2.0	13.7±2.76	*	ns	ns
MPOAa	4.8±1.0	6.1±1.5	0.9±0.3	1.8±0.5	**	ns	ns
MPOAi	4.0±0.6	4.4±0.6	1.0±0.2	1.9±0.2	**	ns	ns
MPOAp	14.3±3.0	13.1±2.9	32.1±6.0	37.4±8.2	**	ns	ns
PVN	530.4±45.6	546.6±48.2	809.6±143.2	828.9±71.1	**	ns	ns

SEM: standard error of the mean;

* *p*<0.05;

** *p*<0.01;

ns, not significantly different.

### TH-ir staining

**Figure 3:**
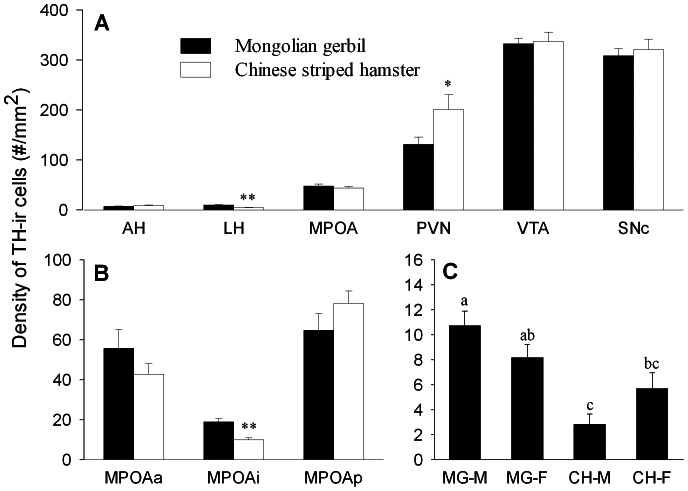
Species differences in the density of TH-ir cells in brain areas. (A) Species differences in the density of TH-ir cells in the anterior hypothalamus (AH), lateral hypothalamus (LH), medial preoptic area (MPOA), paraventricular nucleus of the hypothalamus (PVN), ventral tegmental area (VTA) and substantianigra pars compacta (SNc). (B) Species differences in the density of TH-ir cells in the anterior (MPOAa), intermediate (MPOAi) and posterior (MPOAp) subnuclei of the MPOA. (C) Student-Newman-Keuls (SNK) post hoc test indicated differences in the lateral hypothalamus (LH) between males and females of the two species (MG: Monglian gerbil; CH: Chinese striped hamster; M: male; F: female). Bars with different alphabetic letters differed significantly from each other. * *p*<0.05, ** *p*<0.01.

TH-ir cells were found in many brain areas in both species. For example, dense clusters of TH-ir cells were found in the PVN, VTA and SNc, a moderate cluster of TH-ir cells was found in the MPOA, and scattered TH-ir cells were found in the AH and LH. Species differences were observed (Table 3). Mongolian gerbils had a higher density of TH-ir cells in LH (F_(1,26)_ = 19.07,*p*<0.01) and MPOAi (F_(1,26)_ = 18.52, *p*<0.01), but a lower density of TH-ir cells in the PVN (F_(1,26)_ = 5.21, *p*<0.05), than Chinese striped hamsters (Fig. 3A & B). Sex differences were found in the MPOA (F_(1,26)_ = 9.52, *p*<0.01), particularly in the MPOAa (F_(1,26)_ = 11.06, *p*<0.01) in which females had a higher density of TH-ir cells than males (Table 3). A species-sex interaction was also found in the LH (F_(1,26)_ = 5.28, *p*<0.05) — male Mongolian gerbils had a higher density of TH-ir cells in the LH than male and female Chinese hamsters (Fig. 3C).

**Table 3:** Density of TH immunoreactive cells (mean±SEM/mm^2^) in the brain of male and female Mongolian gerbils and Chinese striped hamsters.

Brain area	Mongolian gerbil		Chinese hamster		Two-way ANOVA		
	Male	Female	Male	Female	species	sex	sp X sex
AH	7.75±1.38	6.90±0.86	7.81±1.44	8.80±2.54	ns	ns	ns
LH	10.73±1.15	8.17±1.06	2.82±0.83	5.67±1.29	**	ns	*
MPOA	38.38±3.14	56.47±6.03	37.71±4.71	49.52±4.96	ns	**	ns
MPOAa	34.01±6.05	77.34±14.76	32.50±7.08	52.98±5.99	ns	**	ns
MPOAi	15.72±2.58	21.93±2.34	9.63±1.53	10.18±1.17	**	ns	ns
MPOAp	65.41±12.11	70.15±12.32	71.01±8.42	85.39±8.75	ns	ns	ns
PVN	129.02±18.12	133.15±24.60	223.12±44.55	180.04±37.44	*	ns	ns
VTA	323.99±16.82	341.02±15.94	342.10±30.30	332.94±22.67	ns	ns	ns
SNc	295.41±15.26	321.44±23.17	328.64±34.89	314.16±24.38	ns	ns	ns

SEM: standard error of the mean;

* *p*<0.05;

** *p*<0.01;

ns, not significantly different.

### ERα-ir staining

In both species, specific patterns of ERα-ir stained cells were found in many forebrain areas. Very intense staining of ERα-ir cells was found in the ARC and MPOA, while moderate clusters of ERα-ir cells were found in the VMH, BST, MeA, CoA and PVN. Scattered ERα-ir cells were found in several brain regions including the AH and LS (Table 4).

**Table 4:** Density of ERα immunoreactive cells (mean±SEM/mm^2^) in the brain of male and female Mongolian gerbils and Chinese striped hamsters.

Brain area	Mongolian gerbil		Chinese hamster		Two-way ANOVA		
	Male	Female	Male	Female	species	sex	sp X sex
AH	78.24±4.81	80.19±5.49	9.85±1.16	17.62±3.05	**	ns	ns
LS	25.35±3.16	20.22±2.97	4.79±1.18	4.07±1.05	**	ns	ns
BST	290.05±21.78	271.70±17.18	162.24±12.94	179.06±19.01	**	ns	ns
MPOA	932.49±58.36	880.42±50.32	1027.7±102.61	914.92±81.05	ns	ns	ns
MPOAa	716.09±152.85	1070.2±174.88	1098.1±233.5	759.6±144.57	ns	ns	ns
MPOAi	520.38±34.16	546.73±29.54	399.92±37.52	451.62±29.46	**	ns	ns
MPOAp	1561.0±178.67	1024.3±167.01	1585.2±158.34	1533.5±267.6	ns	ns	ns
VMH	449.13±19.40	493.65±32.49	275.42±23.46	330.78±39.44	**	ns	ns
ARC	2026.5±158.30	1990.4±170.37	1279.1±186.40	1217.7±117.67	**	ns	ns
MeA	492.34±81.14	475.19±65.65	376.94±32.64	419.24±21.65	ns	ns	ns
CoA	308.55±47.58	362.93±42.79	230.47±14.11	305.48±10.06	ns	ns	ns
PVN	341.05±72.76	309.78±54.44	543.47±53.28	445.26±32.43	**	ns	ns

SEM: standard error of the mean;

** *p*<0.01;

ns, not significantly different.

**Figure 4:**
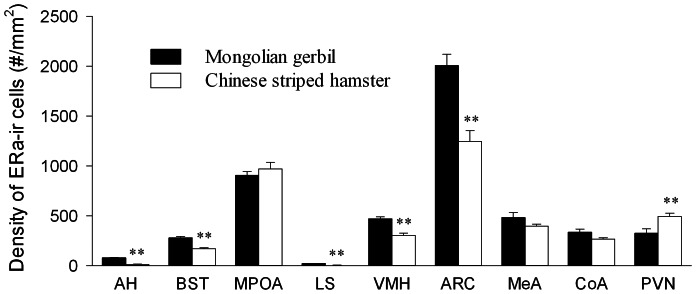
Species differences in the density of ERα-ir cells in brain areas. (A) Species differences in the density of ERα-ir cells in the anterior hypothalamus (AH), bed nucleus of the striaterminalis (BST), medial preoptic area (MPOA), lateral septum (LS), ventral medial hypothalamus (VMH), arcuate nucleus of the hypothalamus (ARC), medial nucleus (MeA) and anterior cortical nucleus (CoA) of the amygdala and paraventricular nucleus of the hypothalamus (PVN). ** *p*<0.01.

Species differences were found in the density of ERα-ir cells in some of the brain areas examined. Mongolian gerbils had a higher density of ERα-ir cells in the AH (F_(1,26)_ = 231.58, *p*<0.01), BST (F_(1,26)_ = 30.76,*p*<0.01), LS (F_(1,26)_ = 53.24, *p*<0.01), VMH (F_(1,26)_ = 32.64,*p*<0.01) and ARC (F_(1,26)_ = 20.18,*p*<0.01) than Chinese striped hamsters (Fig. 4), and a similar species difference was found in the MPOAi (F_(1,26)_ = 10.75,*p*<0.01) (Table 4). In the PVN, however, Chinese hamsters had a higher density of ERα-ir cells than Mongolian gerbils (F_(1,26)_ = 8.85, *p*<0.01). The two species did not differ in the density of ERα-ir cells in the total MPOA, MeA and CoA. Furthermore, no sex differences or species–sex interactions were found in any brain areas examined.

**Figure 5:**
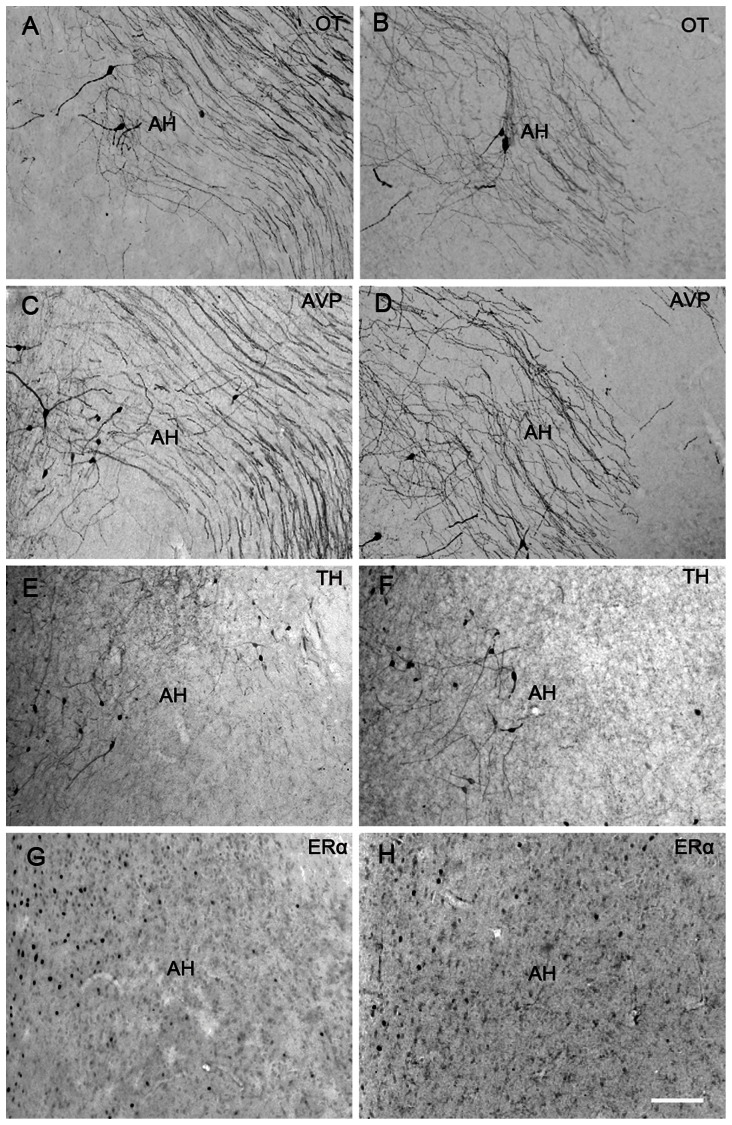
Photomicrographs of neuropeptides expression in the anterior hypothalamus (AH). Photomicrographs displaying OT-ir (A & B), AVP-ir (C & D), TH-ir (E & F) and ERα-ir (G & H) stained cells in the anterior hypothalamus (AH) in the brains of Mongolian gerbils (A, C, E & G), and Chinese striped hamsters (B, D, F & H). Scale bar = 100 µm.

**Figure 6:**
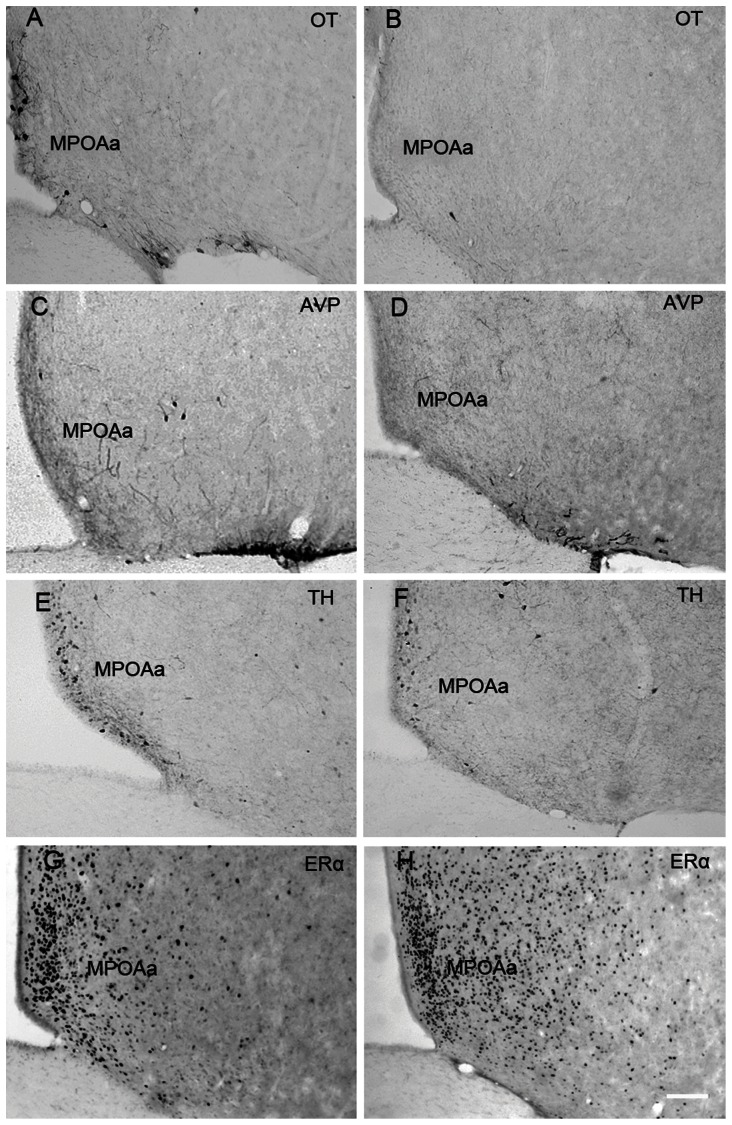
Photomicrographs of neuropeptides expression in the anterior subnucleus of the medial preoptic area (MPOAa). Photomicrographs displaying OT-ir (A & B), AVP-ir (C & D), TH-ir (E & F) and ERα-ir (G & H) stained cells in the anterior subnucleus of the medial preoptic area (MPOAa) in the brains of Mongolian gerbils (A, C, E & G) and Chinese striped hamsters (B, D, F & H). Scale bar = 100 µm.

**Figure 7:**
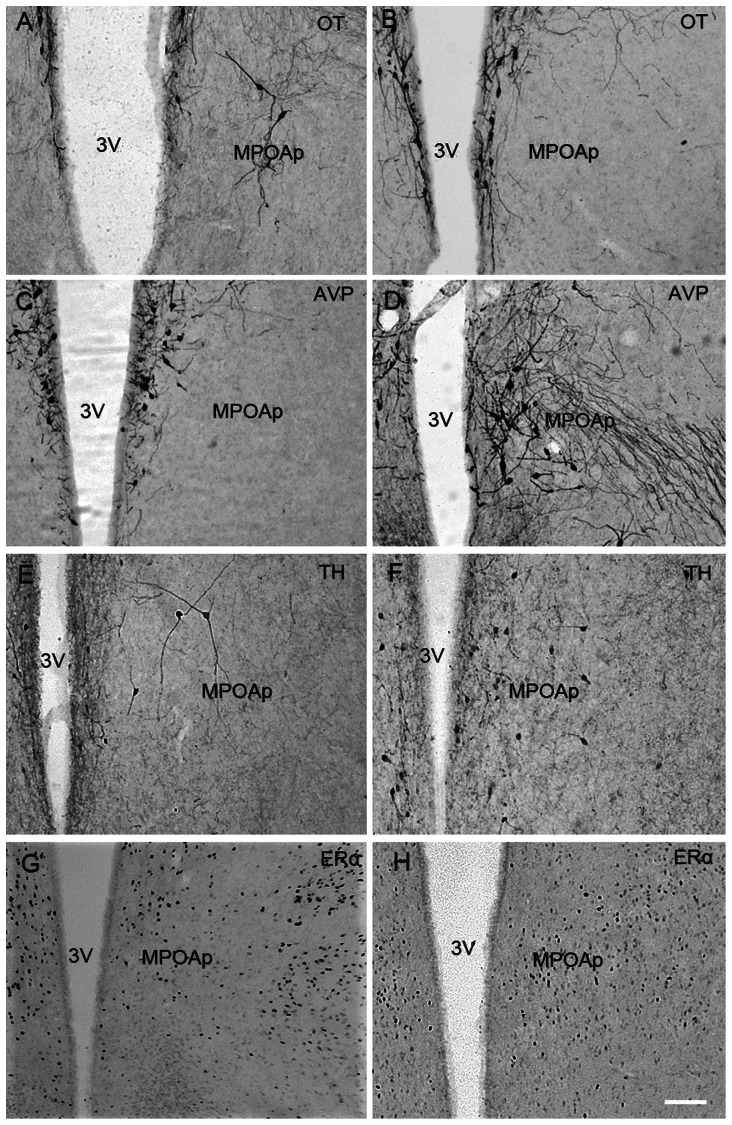
Photomicrographs of neuropeptides expression in the posterior subnucleus of the medial preoptic area (MPOAp). Photomicrographs displaying OT-ir (A & B), AVP-ir (C & D), TH-ir (E & F) and ERα-ir (G & H) stained cells in the posterior subnucleus of the medial preoptic area (MPOAp) in the brains of Mongolian gerbils (A, C, E & G) and Chinese striped hamsters (B, D, F & H). 3V, third ventricle, Scale bar = 100 µm.

Figures 5, 6 and 7 show representative photomicrographs displaying OT-ir, AVP-ir, TH-ir and ERα-ir cells in the AH (Fig. 5), MPOAa (Fig. 6) and MPOAp (Fig. 7) in both species.

## Discussion

Animals with different life strategies usually display different patterns of social behaviors. Previous studies have shown that social Mongolian gerbils display high levels of social behaviors including agonistic behavior, social affiliation and parental care [33], [35], [45]–[48]. These gerbils live in stable family groups consisting of a male and a female, suckling pups and weaning juveniles [33]. The juveniles usually display alloparental care toward their younger siblings [49]. To prepare for winter, all members in the family group participate in food hoarding [50]. In contrast, solitary Chinese striped hamsters are less social and only participate in prosocial contact with conspecifics during mating [41], [42]. In the field, single adult hamsters live alone in burrow nests [41]. A clear distinction has also been reported for their aggressive behavior; Chinese striped hamsters are territorial and display high levels of flank marking behavior and aggression towards conspecifics [41], [42], whereas Mongolian gerbils are less aggressive [33], [35]. In the present study, we compared between these two species, OT, AVP, TH and ERα immunoreactive expression in selected brain areas known to be important in social behaviors. Our data demonstrate species-specific patterns of neurochemical expression in a brain region-specific manner. These data provide further evidence to support the notion that species-specific neurochemical pathways in the brain are associated with and possibly involved in the regulation of social behaviors related to different life strategies (Table 5) [1], [5], [6].

**Table 5:** Neurochemical implications in social behaviors.

Oxytocin				
Behavior	Species	Brain area	Effect	References
Maternal behavior	Rat	BST, MPOA, VTA	↑	[60], [95]
	Naked mole-rat	NAcc, MPOA	↑	[61]
	Prairie vole	NAcc	↑	[96]
	Brandt's vole	MPOA	↑	[2]
	Greater long-tailed hamster	MPOA	↑	[2]
Maternal aggression	Rat	PVN, CeA, BST	↓	[97], [98]
	Syrian hamster	CeA	↑	[99]
Social recognition	Rat	MPOA, LS	↑	[62], [63]
	Mouse	MeA	↑	[100]
	Brandt's vole	MeA	↑	[2]
	Greater long-tailed hamster	MeA	↑	[2]
Pair bonding	Prairie vole	NAcc	↑	[101], [102]
Sexual behavior	Rat	MPOA	↑	[58], [59]

Vasopressin				
Behavior	Species	Brain area	Effect	References
Maternal behavior	Rat	PVN, SON	↑	[103]
Aggression	Prairie vole	AH	↑	[53], [104]
	California mouse	BST, LS	↑	[1]
	White-footed mouse	BST, LS	↑	[1]
	Syrian hamster	AH, VMH	↑	[22], [51], [105]–[107]
Social recognition	Rat	LS	↑	[108], [109]
Pair bonding	Prairie vole	LS	↑	[110]
Flank marking	Syrian hamster	AH, MPOA	↑	[64]

Dopamine				
Behavior	Species	Brain area	Effect	References
Maternal behavior	Rat	NAcc, VTA	↑	[111]–[114]
Maternal aggression	Rat	VTA, Amygdala	↑	[115]
Aggression	Prairie vole	AH	↑	[104], [116]
Pair bonding	Prairie vole	BST, NAcc, MeA	↑	[9], [116]–[118]
Sexual behavior	Rat	NAcc, MPOA	↑	[119]–[121]
Social play	Rat	NAcc	↑	[122]

Estrogen receptor α				
Behavior	Species	Brain area	Effect	References
Maternal behavior	Rat	MPOA	↓	[123]
Aggression	Rat	VMH	↓	[124]
Social recognition	Rat	MeA	↑	[124]
Social affiliation	Prairie vole	BST, MPOA	↓	[11], [16]
Prosocial behavior	Prairie vole	BST, MeA	↓	[11], [14]
	Djungarian hamster	BST	↓	[12]
	Siberian hamster	BST	↓	[12]

↑: increase and↓: decrease in behavior.

In the present study, species differences in neurochemical expression were found in a brain region- and neurochemical-specific manner. In the AH, for example, Mongolian gerbils had higher densities of AVP-ir and ERα-ir, but not OT-ir and TH-ir, cells than Chinese striped hamsters. In the LH, Chinese striped hamsters had a higher density of OT-ir cells, but lower densities of AVP-ir and TH-ir cells, compared to Mongolian gerbils. The AH and LH, as well as neurochemical activity within these regions, have been implicated in a variety of behavioral and physiological functions. For example, AVP and ERα in the AH are involved in flank marking and aggression [15], [51]–[53]. OT and AVP within the LH are involved in feeding and water balance [54]–[57]. It is possible that species differences in the AVP and ERα systems in the AH represent the potential involvement of these neurochemical systems in behaviors related to territory defense. On the other hand, species differences in neurochemical expression within the LH may reflect differences in the central systems regulating behaviors such as feeding and drinking that are important for maintaining one's homeostasis [54]–[56]. Although little is known about feeding and drinking behaviors in these two species, these animals live in distinct geographical regions with different ecological and environmental conditions [35], [41]. Therefore, the species differences in central neurochemical systems noted above may reflect physiological, in addition to behavioral, adaptations to the environment.

The MPOA is a brain area important for a large variety of social behaviors including mating [58], [59], maternal care [60], [61], social recognition [62], [63], territory marking [64] and aggression [65]–[67]. This is a complex brain structure consisting of several subnuclei [2], [68]–[70]. Unfortunately, we know very little about the structural and functional significance of these subnuclei within the MPOA. An interesting finding in the present study is that Mongolian gerbils had higher densities of OT-ir and AVP-ir cells in the MPOAa compared to Chinese striped hamsters, whereas an opposite pattern was found in the MPOAp for both neuropeptides. These data are generally consistent with the data from a previous study comparing social Brandt's voles with solitary Greater long-tailed hamsters [2]. One possibility is that the MPOAa and MPOAp, as well as neuropeptide activity within these regions, have opposing effects on behavioral and physiological functions associated with a social or solitary life strategy. In a previous study in rats, microinjections of an anti-androgen drug into the anteroventral MPOA decreased copulatory behavior but had no effects on sexual motivation, whereas microinjections of the same drug into the posterodorsal MPOA did not influence copulatory performance but decreased sexual motivation [71].

Our data also indicate sexually dimorphic patterns of neurochemical expression in the brain. The density of OT-ir cells in the MPOAi was significantly higher in females than in males in both species. Furthermore, the density of TH-ir cells was higher in the MPOA, particularly in the MPOAa, in females than in males in both species. Discrepancies have been found in previous studies in other rodent species focusing on the MPOA. For example, in rats, the anteroventral periventricular nucleus of the preoptic area (AVPV), which is a sub-region of MPOA, is larger in females than in males [72]. In mice, however, males have a larger MPOA than females [73]. In both rats and mice, females have more TH-expressing cells in the AVPV than males [72], [74]–[77]. However, no sex differences are found in the number of OT-ir cells in the MPOA in voles [2], [28], [61]. Furthermore, although female rats and voles have more cells labeled for ERα in the MPOA and other brain areas (e.g., the VMH, BNST and MeA) [11], [78] compared to males, we did not observe such sex differences in the two species examined in the present study. Therefore, sexually dimorphic patterns of OT-ir and TH-ir expression in the MPOA could be species-specific. Region-specific neurochemical expression is involved in behavioral functions. For example, OT in the MPOA is involved in mating behavior [58], [59], maternal care [60], [61] and social recognition [62], [63]. The functional roles of the sexually dimorphic neurochemical expression noted in the two species examined here are still unknown.

It is worth mentioning that the neurochemicals examined in the present study may interact to influence each other's expression and functions. For example, it has been well documented that estrogen can directly influence the expression and activity of central AVP and OT systems [79], [80]. Estrogen can up-regulate AVP expression in several brain areas including the LS, BST, AH and amygdala [20], [21], [79]. Estrogen can also regulate OT expression in the PVN [80], [81] and OT receptor binding in the MPOA [82]. Further, TH-ir neurons in the PVN are found to co-express AVP [83], and thus dopamine may influence local AVP expression and release [84]. Therefore, it is not surprising to see similar species differences in multiple neurochemicals in a given brain area, and in fact, synergistic effects of multiple neurotransmitters on behavior have been amply demonstrated [85], [86].

Some caveats in the present study need to be discussed. First, while Mongolian gerbils were the offspring of a laboratory breeding colony and were sexually naïve and age matched, Chinese striped hamsters were field captured and thus their ages and reproductive history were unknown. It is worth mentioning that the expression patterns in the brain of the neurochemicals under investigation have been shown to change not only during development but also as a result of reproductive experience and aging [2], [87]. Second, females of both species in the present study are spontaneous ovulators [88], [89]. The fluctuation of circulating estrogen during ovarian cycles is known to influence brain neurochemical expression, including OT [90], [91], AVP [20], [21] and TH [87], as well as to alter its own receptor expression [43], [92], [93]. Therefore, potential variations in circulating levels of estrogen among the females might have affected the observed presence or absence of sexual dimorphisms in neurochemical expression in the present study, as has been reported for typical lab rodent species [43], [94]. Finally, our subject's housing conditions reflected their species-specific life strategies. It is possible, however, that different degrees of social experience during housing may have affected the observed neurochemical staining. Although beyond the scope of the present experiment, these issues need to be considered in follow-up studies.
